# Pseudothrombotic microangiopathy resulting from severe vitamin B12 deficiency in a pediatric patient with autism: A case report

**DOI:** 10.1177/2050313X251377265

**Published:** 2025-09-25

**Authors:** Ilana Walters, Caroline Malcolmson, Renee Potashner, Cassandra Nelson, Romy Cho

**Affiliations:** 1 Department of Paediatrics, University of Toronto, Toronto, ON, Canada; 2 Division of Paediatric Medicine, The Hospital for Sick Children, Toronto, ON, Canada; 3 Division of Haematology and Oncology, The Hospital for Sick Children, Toronto, ON, Canada

**Keywords:** pseudothrombotic microangiopathy, autism spectrum disorder, vitamin B12 deficiency, pediatric, case report

## Abstract

Vitamin B12 deficiency is typically characterized by megaloblastic anemia, but can also rarely present with pseudomicroangiopathic hemolytic anemia or multilineage cytopenias secondary to dyserythropoiesis and apoptotic cell death. We describe a 12-year-old male with autism spectrum disorder who presented with fever, night sweats, and weight loss. Laboratory testing identified severe neutropenia and hemolytic anemia, and an undetectably low vitamin B12 level. Dietary history confirmed significant food restriction over the preceding 6 months. He received 1000 µg of intramuscular vitamin B12 daily for 7 days and subsequently transitioned to oral therapy. His hemoglobin, hemolytic markers, and neutrophil count normalized within 13 days of vitamin B12 initiation. Our report describes a rare and severe manifestation of vitamin B12 deficiency in a patient with autism spectrum disorder and emphasizes the importance of screening for micronutrient deficiencies among vulnerable pediatric populations to avoid unnecessary and invasive testing.

## Introduction

Feeding difficulties are observed in 46%–89% of children with autism spectrum disorder (ASD)^1^ and are often characterized by restrictive eating behaviors with food selectivity and/or refusal.^1,2^ The etiology of feeding restriction in children with ASD is multifactorial, including behavioral challenges with mealtimes, abnormal sensory processing, oromotor difficulties, and/or gastrointestinal problems.^2^ As a result, children with ASD are at increased risk of nutritional deficiencies.^3,4^

There is limited literature describing the true prevalence of micronutrient deficiencies among children with ASD. In a narrative review by Daniel et al., 44 children were identified on review of case reports and case series reporting micronutrient deficiencies in children with ASD published between 2014 and 2025.^5^ Approximately 8% of these cases were diagnosed with vitamin B12 deficiency with additional screening after another micronutrient deficiency was identified with no associated sequelae. One case reported optic neuropathy with progressive vision loss related to low vitamin B12.^5^ Vitamin B12 plays a critical role in neurodevelopment, through its roles in the synthesis of serotonin and dopamine as well as neuronal myelination.^6^ Emerging research has demonstrated that people with ASD often have alterations in their vitamin B12 levels, with further studies required to determine if there is a causal relationship between vitamin B12 levels and severity of disease.^6^ This case report describes a pediatric patient with ASD who presents with a rare and severe presentation of vitamin B12 deficiency.

## Case report

A 12-year-old fully vaccinated nonverbal male with ASD presented to a pediatric hospital emergency room with a 1-week history of fever associated with 1 month of night sweats and progressive weight loss over the prior 6 months. On initial assessment, vital signs were stable, and the physical examination was grossly unremarkable with no lymphadenopathy or hepatosplenomegaly. Initial laboratory testing reported severe neutropenia and anemia. The patient was subsequently admitted for further medical work-up and consultation by the Pediatric Hematology–Oncology specialists.

**Figure 1.**
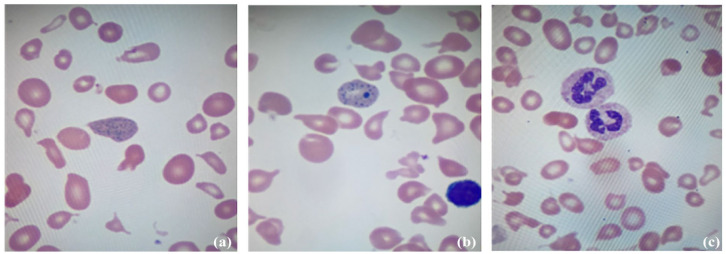
Patient’s peripheral blood smear. Red cells are irregular and abnormally shaped (poikilocytosis). Basophilic stippling (a, b) and hypersegmented neutrophils (c) are present.

During admission, the patient had intermittent fevers with stable vital signs. The highest fever of 38.9 °C (10 was at home prior to admission). Repeat laboratory investigations documented neutropenia of 0.34 × 10^9^/L (reference range: 1.45–6.75 × 10^9^/L), a hemoglobin of 52 g/L (reference range: 112–141 g/L), a normal mean corpuscular volume (MCV) of 89.3 fL (reference range 77.6–91.0) and an inappropriately low reticulocyte count of 25 g/L (reference range: 41.6–65.1 × 10^9^/L). There was evidence of hemolysis, including elevated unconjugated bilirubin, elevated LDH, and an undetectably low haptoglobin. His peripheral blood smear showed basophilic stippling, hypersegmented neutrophils, poikilocytosis, and schistocytes (Figure 1). His platelet count of 174 × 10^9^/L was at the lower end of normal (reference range: 173–361 × 10^9^/L). His liver and renal function tests were normal. On detailed medical history, the patient was described to have had picky eating behaviors for several years. However, caregivers had noted a profound, gradual decrease in the types of food and portions he would eat over the preceding 6 months, which was associated with at least 7.2 kg (14%) loss in body weight. The patient had experienced a social stressor around the onset of this change, which may have contributed to his decreased intake. At the time of presentation, he was only consuming small amounts of toast, fruit juice, crackers, and potato chips.

Based on the history of restricted intake, testing was completed to assess for nutritional deficiencies. The patient’s vitamin B12 level was undetectably low at <61 pmol/L (reference range: 186–830 pmol/L) and his iron studies were consistent with iron overload with a serum iron level of 29.3 µmol/L (reference range: 4.8–25.3 µmol/L), serum transferrin level of 22.4 µmol/L (reference range: 27.1–41.5 µmol/L), and serum ferritin level of 341.2 µg/L (reference range: 3.7–78.8 µg/L), likely secondary to chronic hemolysis. The patient was also documented to have a low vitamin D level of 36 nmol/L (reference range: 70–250 nmol/L). The remainder of his testing for nutritional deficiencies was grossly normal (Table 1). Other causes of vitamin B12 deficiency, including pernicious anemia, inflammatory bowel disease, and celiac disease, were all investigated and excluded.

**Table 1.** Initial laboratory results assessing for nutritional deficiencies in the patient.

Investigation	Patient’s initial result	Reference range
Serum iron	**29.3**	4.8–25.3 µmol/L
Transferrin	**22.4**	27.1–41.5 µmol/L
Serum ferritin	**341.2**	13.7–78.8 µg/L
Serum folate	33.4	>27.0 nmol/L
Vitamin B12	**<61**	186–830 pmol/L
Vitamin E	28	12–31 µmol/L
Vitamin A	**0.9**	1.0–2.6
Vitamin D	**36**	70–250 nmol/L
Ascorbic acid	88	⩾25 µmol/L

Test results with values outside of reference range have been bolded.

Given the presence of constitutional symptoms, concurrent initial screening for an oncologic etiology was undertaken. The patient completed a chest X-ray to evaluate for mediastinal mass, an abdominal ultrasound to evaluate for lymphadenopathy and masses, a flow cytometry to screen for leukemia, and an ultrasound of the neck to evaluate the architecture of lymph nodes. All of these investigations were normal.

The patient was diagnosed with severe vitamin B12 deficiency with secondary pseudomicroangiopathic hemolytic anemia and neutropenia. He was initially started on 1000 µg of intramuscular vitamin B12 daily for 7 days and transitioned to oral therapy. His vitamin B12 level rapidly normalized within 24 h of initiating treatment. His reticulocytes began to trend up within 48 h of initiating therapy. His hemoglobin, hemolytic markers, and neutrophil count normalized within 13 days of vitamin B12 initiation. At the time of discharge, he was afebrile with significant improvement in appetite and energy. He was discharged home on daily vitamin B12 1000 µg, vitamin D 1000 IU, and a multivitamin supplement.

The patient had regular bloodwork monitoring and follow-up with the Pediatric Nutrition clinic for 2 years with no adverse events. He was compliant with vitamin B12; however, vitamin D was modified from daily tablets to drops taken once weekly, and the multivitamin supplement was changed to a tasteless solution. He regained the weight he had lost and is followed by a developmental pediatrician affiliated with a children’s treatment center.

## Discussion

Vitamin B12 deficiency may present clinically with fatigue, pallor, weight loss, paresthesia, weakness, and ataxia.^7^ Vitamin B12 deficiency can be caused by decreased dietary intake, malabsorption, or secondary to an autoimmune process.^8^ Dietary deficiency is typically related to decreased dietary intake of animal, dairy, or egg products and is more likely to be seen in children with restrictive diets.^8^ Malabsorption occurs when absorption is impacted at the levels of either the gastric mucosa or the terminal ileum secondary to gastric bypass surgery, *Helicobacter pylori* infection, or inflammation in the terminal ileum, such as in inflammatory bowel disease.^9^ Finally, an autoimmune process, such as pernicious anemia, can cause autoantibodies to bind to intrinsic factor and inhibit vitamin B12 from being absorbed through the gastric mucosa.^10^

Pseudothrombotic microangiopathy (pseudo-TMA) is a rare presentation of vitamin B12 deficiency. Patients typically develop thrombocytopenia and anemia, with laboratory findings of schistocytes on peripheral smear and elevated hemolytic markers. It is important to differentiate this from other causes of TMA, such as thrombotic thrombocytopenic purpura (TTP) or hemolytic uremic syndrome. In a single-center retrospective analysis of 13 adult patients presenting with the above features, patients with pseudo-TMA had higher platelet counts, lower neutrophil counts, lower reticulocyte counts, and higher LDH than those with TTP.^11^ A similar metabolism-mediated TMA occurs in cobalamin C disease, a hereditary defect in vitamin B12 metabolism.

Classically, vitamin B12 deficiency presents with laboratory findings of megaloblastic anemia, characterized by low hemoglobin and an increased MCV. Less commonly, patients may present with leukopenia, thrombocytopenia, and rarely pancytopenia and hemolysis because of ineffective DNA synthesis.^7^ In our case, there was diagnostic complexity as our patient presented with a normocytic anemia, which is atypical in vitamin B12 deficiency. This highlights that MCV can be a misleading parameter, especially in the setting of underlying hemoglobinopathies, hemolysis, or concurrent micronutrient deficiencies (e.g. a combined iron and vitamin B12 deficiency). Given the vague systemic complaints and the varied hematologic manifestations, it is important to have a high index of suspicion for vitamin B12 deficiency, as severe disease can mimic acute leukemia, which can lead to additional investigations, such as bone marrow biopsy and aspiration.^12^ With the early and prompt recognition of the vitamin B12 deficiency based on dietary history and consistent lab findings including macrocytosis, basophilic stippling, and hypersegmented neutrophils on peripheral smear, a step-wise approach to the investigations was undertaken in this case. While there is paucity in the literature to explain the presence of constitutional symptoms in the setting of severe vitamin B12 deficiency, patients with a similar presentation often underwent bone marrow evaluation, which aided in the diagnosis of vitamin B12 deficiency.^12–14^ With a high suspicion for vitamin B12 deficiency, concurrent screening for an oncologic etiology can begin with less invasive testing, including imaging and peripheral flow cytometry. In the absence of concerning features with these investigations, the decision to undergo more definitive bone marrow testing could be made based on the response to vitamin B12 replacement therapy. In this case, since there were no concerning features from radiologic and peripheral flow cytometry testing, and with clinical and laboratory improvements in his blood counts following the administration of vitamin B12, there was no additional indication to proceed with bone marrow biopsy testing. In cases where constitutional symptoms or pancytopenia do not improve as would be expected with vitamin B12 replacement, more definitive testing with bone marrow aspirate and biopsy would be indicated.^15^

Children with ASD are at increased risk of nutritional deficiencies^3,4^ and case reports have described micronutrient deficiencies in children with ASD who have experienced invasive testing, prolonged hospital admissions, and delayed diagnoses.^4^ Esposito et al. advocates for regular assessment of anthropometric data, dietary information using a food diary, and eating behaviors in children with ASD^16^ and these recommendations are more broadly applicable to other pediatric populations with restrictive eating patterns or impaired absorption, for example, patients with chronic gastrointestinal conditions,^9^ eating disorders,^17^ food scarcity,^18^ or children with neurological impairment and oral motor challenges.^19^

## Conclusion

Our case describes a rare and severe presentation of vitamin B12 deficiency in a pediatric patient with ASD. The spectrum of clinical symptoms and laboratory findings can lead to over-investigation, as well as delay diagnosis and treatment; therefore, it is prudent to proactively assess and screen for nutritional deficiencies in the pediatric population, particularly among patients at higher risk.
